# Surgical Perspectives on Neoadjuvant Therapy in Borderline Resectable and Locally Advanced Pancreatic Cancer

**DOI:** 10.3390/cancers18071131

**Published:** 2026-04-01

**Authors:** Jingcheng Zhang, Menghang Geng, Helmut Friess, Ihsan Ekin Demir, Florian Scheufele

**Affiliations:** Department of Surgery, Klinikum Rechts der Isar, School of Medicine and Health, Technical University of Munich, 81675 Munich, Germany; jingcheng.zhang@tum.de (J.Z.);

**Keywords:** pancreatic ductal adenocarcinoma, neoadjuvant therapy, borderline resectable, locally advanced, conversion surgery

## Abstract

**Background/Objectives**: Neoadjuvant therapy (NAT) is now central to the management of borderline resectable (BRPC) and locally advanced (LAPC) pancreatic ductal adenocarcinoma (PDAC). This narrative review summarizes contemporary evidence and guidelines from a surgical perspective, with emphasis on pretreatment classification, post-NAT selection for exploration, intraoperative vascular strategy, and postoperative management. **Methods**: We conducted a structured narrative review of randomized and prospective studies, high-quality observational cohorts, and major international guidelines published through 31 July 2025. **Results**: BRPC and LAPC remain primarily defined by vascular anatomy, but biologic and conditional factors are increasingly integrated into decision-making. NAT is the preferred initial strategy for BRPC and the standard induction approach for LAPC, with resection considered only in carefully selected responders. After NAT, contrast-enhanced CT combined with CA19-9 kinetics remains the core restaging platform, while FDG-PET, diffusion-weighted MRI, radiomics, and circulating biomarkers may serve as adjuncts in equivocal cases. Surgical exploration should be guided by physiologic recovery, the absence of metastatic progression, and multidisciplinary reassessment. Staging laparoscopy remains useful for detecting occult metastatic disease. Intraoperatively, vascular resection should be margin-driven rather than routine, with portal–mesenteric venous resection established in expert centers, whereas arterial resection remains highly selective. Periarterial divestment represents an artery-sparing alternative in selected cases. NAT does not appear to worsen short-term postoperative outcomes, but anticoagulation after venous reconstruction remains non-standardized. **Conclusions**: NAT has transformed BRPC/LAPC PDAC into a biology-gated, time-sequenced surgical pathway. Standardized reassessment, careful candidate selection, and the centralization of complex vascular procedures are essential to optimize outcomes.

## Simple Summary

Pancreatic cancer that extends to major blood vessels is difficult to treat and often cannot be managed by upfront surgery alone. Preoperative treatment is increasingly used to improve tumor control, select patients with more favorable disease biology, and increase the chance of complete resection. However, major challenges remain in defining which patients should undergo surgery after treatment and how vascular involvement should be managed intraoperatively. In this review, we summarize current evidence and guideline-based practice from a surgical perspective, focusing on patient selection, restaging, surgical exploration, and vessel resection strategies. By integrating oncologic, radiologic, and operative considerations, this review provides a practical framework for decision-making in patients with borderline resectable or locally advanced pancreatic cancer. These insights may support more standardized clinical practice and help guide the design of future studies in this evolving field.

## 1. Introduction

Pancreatic cancer (PC) is one of the few common solid malignancies with mortality still rising. It ranks third among causes of cancer-related death, and the overall 5-year relative survival remains below 10%. For pancreatic ductal adenocarcinoma (PDAC), which accounts for 90% of cases, the 5-year survival falls below 8% [1]. A similar pattern is seen in Germany, where PC is the fourth leading cause of cancer mortality in both sexes and the 5-year survival is around 11% [2].

Although resection followed by multi-agent adjuvant therapy offers the only chance of cure [3], this option is available to merely 15–20% of patients, whereas around half present with distant disease at diagnosis, and a substantial proportion are diagnosed with borderline resectable pancreatic cancer (BRPC) or locally advanced pancreatic cancer (LAPC) [1,2,4]. In BRPC or LAPC, upfront surgery is often associated with inferior outcomes due to the technical difficulty of achieving an R0 resection in the setting of vascular involvement and the high risk of occult micro-metastatic disease that cannot be addressed by surgery alone [5,6]. Against this backdrop, neoadjuvant therapy (NAT) has become integral to the management of BRPC and an increasingly considered option for LAPC.

As NAT has moved into routine practice, the central challenge has shifted from whether to treat preoperatively to how to make consistent, defensible surgical decisions after treatment. Restaging often yields imperfect and sometimes discordant signals from imaging, biomarkers, and clinical recovery; operative planning must account for venous and arterial strategies in patients whose tumors remain tethered to major vessels, and perioperative management increasingly requires explicit balancing of thrombotic and bleeding hazards, particularly when vascular reconstruction is performed. Current guidelines outline broad principles but leave substantial variability in implementation across centers.

In this narrative review, we synthesize contemporary guidance and evidence on NAT for BRPC and LAPC through a surgical lens and translate it into a practical decision framework spanning pretreatment stratification, post-NAT reassessment, intraoperative vascular strategy, and perioperative risk management.

## 2. Materials and Methods

We conducted a narrative review using a structured search strategy rather than a formal systematic review. PubMed was searched from inception to 31 July 2025 for peer-reviewed, English-language, human studies with MeSH and free-text terms covering pancreatic neoplasms, neoadjuvant or preoperative therapy, borderline resectable or locally advanced disease, surgery, and vascular management. Eligible evidence comprised randomized trials, prospective/retrospective cohorts, registries, meta-analyses, and society guidance/consensus documents that directly inform decision-making about NAT in adults with BR/LA PDAC. Case reports, editorials, letters, and abstracts were excluded due to limited methodological transparency and lack of peer review. The evidence was synthesized with attention to study design and relative level of evidence. As a narrative review, this article does not provide a formal risk-of-bias assessment, which should be considered when interpreting the summarized evidence.

## 3. Pretreatment Classification

The designation of BRPC or LAPC is anchored in the anatomic interface between the primary tumor and major vessels, notably the superior mesenteric artery (SMA), celiac axis (CA), common hepatic artery (CHA), portal vein (PV), and superior mesenteric vein (SMV). Although specific cut-offs differ among guidelines [7], the NCCN definitions are widely used in surgical practice [8]. To mirror real-world complexity, the MD Anderson group was the first to extend BRPC beyond anatomy by adding biologic factors, such as elevated CA19-9, and conditional factors, such as performance status and comorbidity, thereby identifying anatomically resectable patients who may still benefit from NAT because of systemic risk or limited tolerance for surgery [9]. These refinements aimed to identify patients who, despite anatomical resectability, may benefit from preoperative therapy due to a higher risk of systemic progression or postoperative intolerance. The International Association of Pancreatology (IAP) subsequently codified a three-domain framework in 2017 that includes: (1) anatomical involvement of adjacent vasculature, (2) biological risk, defined by CA19-9 level > 500 U/mL or regional lymph node metastasis confirmed by biopsy or PET-CT, and (3) patient-related considerations, such as an Eastern Cooperative Oncology Group (ECOG) performance status of ≥2 [10]. This framework has since been incorporated into the treatment algorithms of organizations such as the European Society for Medical Oncology (ESMO) [11]. While NCCN does not formally incorporate biologic or conditional domains into BRPC categorization, it emphasizes performance status as a central determinant of treatment selection [8].

## 4. Who Should Receive NAT?

Current guidelines are broadly concordant regarding when NAT should be used in BRPC and LAPC, while differing in emphasis and in how strongly recommendations are linked to randomized evidence (Table 1). For BRPC, NAT is the preferred initial approach across NCCN (v2.2025) [8], ASCO guideline updates for potentially curable disease [12,13], and the Japanese Pancreas Society (JPS) [14], while ESMO (2023 guideline/2024 commentary) [11] and CSCO [15] likewise support NAT, with a stronger emphasis on clinical-trial participation when feasible. Across these guidelines, multi-agent chemotherapy backbones—including FOLFIRINOX/mFOLFIRINOX and gemcitabine plus nab-paclitaxel (GnP)—are typically listed as preferred options, but no guideline endorses a single neoadjuvant standard regimen, sequencing, or duration. For LAPC, recommendations converge on induction systemic chemotherapy with selective use of chemoradiotherapy, and on considering exploration or resection only for responders after multidisciplinary review, with a consistent preference for trial participation. Importantly, many regimen recommendations in LAPC are extrapolated from metastatic-disease trials rather than from head-to-head neoadjuvant comparisons, reinforcing the absence of a universally accepted neoadjuvant standard.

Since 2018, randomized evidence has increasingly supported NAT over immediate surgery in BRPC when the objective is survival and intention-to-treat oncologic endpoints rather than regimen superiority [16,17,18,19]. For instance, in the ESPAC-5 phase II trial, short-course NAT (eight weeks) significantly improved 1-year overall survival (OS) versus immediate surgery: 39% with immediate surgery, 78% with gemcitabine–capecitabine, 84% with FOLFIRINOX, and 60% with capecitabine-based CRT [18]. Long-term results from PREOPANC showed that, despite a slight improvement in median OS (15.7 vs. 14.3 months), gemcitabine-based NAT yielded a substantially higher 5-year OS (20.5% vs. 6.5%) and increased intention-to-treat R0 rates (41% vs. 28%) compared with upfront surgery [19]. A 2025 meta-analysis pooling four BRPC RCTs plus one mixed BRPC/resectable study (BRPC subgroup *n* = 677) reported an OS advantage for NAT in BRPC (HR 0.60, 95% CI 0.38–0.96), with no clear benefit in clearly resectable disease—reinforcing BRPC as the principal indication for NAT [20].

For LAPC, randomized evidence remains limited, yet prospective signals show that neoadjuvant induction can make surgery possible in a selected subset of patients [21,22]. In LAPACT, induction GnP demonstrated activity and tolerability and allowed some patients to proceed to resection, supporting a conversion-oriented pathway [21]. In the NEOLAP cohort, roughly one third of patients underwent complete macroscopic resection after induction, although a survival advantage was not demonstrated, indicating that efficacy signals require confirmation [22]. In parallel, retrospective data consistently associate conversion surgery with longer survival. A 2022 meta-analysis of 125 studies including 11,713 patients estimated a pooled resection rate of 22.2% for LAPC and reported median overall survival of 30.0 months with resection versus 14.6 months without resection [23]. Most recently, the multicenter PC-CURE-1 cohort showed median overall survival of 34.4 months after surgery versus 19.8 months without surgery, with a hazard ratio for death of 0.47 favoring resection [24]. Collectively, these data support a conversion surgery strategy for carefully selected responders after MDT review, while explicitly acknowledging the selection bias inherent to nonrandomized cohorts and the need for randomized confirmation of survival benefit.

***Practical takeaway***. In current practice, NAT should be considered the default strategy for most patients with BRPC and for selected patients with LAPC who remain fit for multimodal treatment. The decision should not rely on vascular anatomy alone, but should integrate anatomic extent, biologic behavior, and physiologic condition. In this framework, NAT serves not only as treatment, but also as a selection tool for subsequent exploration.

## 5. Post-NAT Response Assessment—Who Proceeds to Exploration?

Assessing response after NAT is crucial for triaging true surgical candidates and avoiding nonbeneficial exploration. The primary prerequisite is the patient’s conditional recovery—performance status, resolution of treatment-related toxicity, and nutritional reserve (including sarcopenia)—which carries clear prognostic significance and should gate any operative intent [25,26,27]. On the oncologic side, decision-making relies on an integrated interpretation of cross-sectional imaging and biologic markers to support a pragmatic go/no-go determination.

### 5.1. Conventional Computd Tomography (CT)

Contrast-enhanced CT remains the backbone of post-NAT restaging in contemporary guidelines and routine practice. Yet morphologic CT has limited accuracy for predicting resectability and margin status, due to therapy-induced fibrosis and vascular remodeling [28,29]. A 2021 meta-analysis illustrates the imprecision for R0 prediction: Under a strict resectable-only definition (*n* = 217), sensitivity was only 45% despite a specificity of 85%; when borderline resectable cases were included (*n* = 160), sensitivity rose to 81% but specificity dropped to 42% [30]. In the centrally reviewed NEOLAP imaging cohort, tumor size reduction performed better than categorical resectability labels: Any downsizing achieved high sensitivity for predicting R0 (97%; NPV 0.88), and an exploratory 22.5% shrinkage threshold balanced test characteristics [31]. Collectively, these data support interpreting CT findings in context and caution against using the absence of radiographic regression as a sole reason to withhold exploration.

### 5.2. Advanced CT Techniques

Multiple CT-based innovations spanning radiomics and delta radiomics, dual energy CT (DECT) iodine mapping, and quantitative CT perfusion appear to outperform morphology alone for post-NAT assessment [32]. In a 2024 cohort (*n* = 86), post-chemotherapy CT radiomics classifiers predicted favorable pathological response, with the best model reaching an AUC of 0.923 on the held-out test set—substantially above standard reads [33]. An externally validated delta-radiomics study (discovery *n* = 58; validation *n* = 31) showed that combining pre/post-NAT radiomic changes with clinical variables improved prediction of margin status and survival endpoints versus clinical models alone [34]. A larger analysis (*n* = 122; independent test *n* = 25) reported that a core plus edge delta-radiomics model for therapy response achieved AUC 0.899 in training and 0.940 in testing [35]. Beyond texture features, extracellular volume fraction from DECT iodine maps tracked CA19-9 change during chemoradiation, and small single-center series reported AUCs around 0.80 for discriminating pathological responders after preoperative chemoradiation [36]. Quantitative CT perfusion also shows promise for therapy response assessment, though protocols and effect sizes vary and standardization is needed [37]. At present, these techniques are best framed as adjunctive risk stratification tools, requiring harmonized acquisition/segmentation and prospective multicenter validation before routine decision-making adoption.

### 5.3. Diffusion-Weighted MRI (DWI)

The feasibility of DWI for response assessment remains uncertain. A recent systematic review and meta-analysis of seven studies (*n* = 161) concluded that pre/post-NAT apparent diffusion coefficient (ADC) values substantially overlap between responders and non-responders. Although a few small cohorts reported high sensitivities (92–100%) with variable specificities (63–95%), heterogeneity and risk of bias preclude firm recommendations [38]. More recent primary data are likewise divergent: In BRPC (*n* = 72), a multiparametric DWI model identified change in ADC as an independent predictor of treatment response (AUC = 0.94) [39], whereas a larger retrospective cohort (*n* = 103) showed post-NAT ADC increases of approximately 20% to 25% that did not predict R0 status or tumor regression grade [40]. Accordingly, DWI is currently best considered a selective adjunct rather than a routine determinant of operability.

### 5.4. FDG-PET

Across contemporary studies, metabolic response on FDG-PET tracks viable tumor burden and outperforms morphology alone for prognostication. In a large single-center series of resected BR/LA PDAC (*n* = 202), post-NAT metabolic response was the strongest preoperative predictor of major pathologic response (AUC 0.86 vs. 0.75 for CA19-9) and independently predicted recurrence-free survival (RFS) and OS [41]. Complementary evidence shows that adding FDG-PET/MRI (±contrast-enhanced CT improves post-NAT resectability prediction (higher sensitivity and overall AUC) compared with CT alone, and PET-based metrics contribute to survival modeling after R0 resection [42]. Furthermore, emerging analyses further suggest that total lesion glycolysis (TLG) may be a superior metabolic biomarker among PET parameters for forecasting treatment response and prognosis following NAT. Taken together, FDG-PET provides a biologically anchored complement to CT/MRI and serum markers and is likely most useful in biologically or radiologically equivocal cases, although its role as a routine gatekeeper for exploration remains to be defined.

### 5.5. Biological Marker

CA19-9 is the most widely used biologic marker for response assessment after NAT in PDAC and is explicitly incorporated into contemporary guideline frameworks for post-NAT assessment and surgical decision-making. Both NCCN and ESMO recommend integrating CA19-9 decrease or normalization with cross-sectional imaging and clinical status when determining operability after NAT. A 2025 meta-analysis including 92 studies from 2010 to 2024 of resected, localized PDAC found that CA19-9 normalization after NAT is among the strongest prognostic factors for overall survival, alongside margin and nodal status, reinforcing its use in preoperative risk stratification [43]. Interpretation, however, is context-dependent. Recent Japanese cohorts consistently show that post-NAT CA19-9 <100 U/mL independently predicts longer survival [44]. A 2024 multicenter retrospective study (*n* = 492) developed a composite CA19-9 score and derived data-driven cut-offs of 202 U/mL pre-NAT and 78 U/mL post-NAT for overall survival discrimination, with worsening outcomes when CA19-9 rose during therapy [45]. Beyond absolute cut-offs, the percentage decline in CA19-9 can also serve as a response metric. In LAPC, multicenter analyses have proposed a ≥40% early drop (around 2 months) as a minimally meaningful threshold, with deeper falls ≥60% associated with greater benefit [46]. Similarly, a 2025 surgical-oncology cohort stratified “favorable responders” by normalization or post-NAT <296 U/mL plus >40% reduction, demonstrating clear survival separation among patients with high-baseline CA19-9 [47]. Conversely, persistently high post-NAT levels mark adverse biology: A large 2024 cohort (*n* = 609) associated striking post-therapy elevations with distinctly poor prognosis, and postoperative re-rise after prior normalization portended early recurrence [48]. In addition, the Japanese work referenced above compared neoadjuvant chemotherapy (NAC) with neoadjuvant chemoradiotherapy (NACRT) and found that the prognostic significance of post-NACRT CA19-9 values was inferior to that of post-NAC CA19-9, underscoring context-specific behavior of this biomarker across treatment modalities. These observations also highlight current gaps—non-standardized sampling windows, cut-point derivation, and regimen-specific variability, reinforce the need for prospective, standardized, and regimen-stratified studies.

Beyond CA19-9, circulating tumor DNA (ctDNA) is increasingly recognized as a diagnostic and prognostic biomarker in PDAC [49,50]. For CA19-9 non-secretors, CA125 and CEA are useful adjunct serum markers and are recommended for routine measurement in this subgroup [8,51]. Regarding post-NAT response assessment, evidence remains limited. Recently, a prospective study (*n* = 84) showed that on-treatment clearance of mutant KRAS ctDNA was associated with longer overall survival, and the persistence of KRAS G12V predicted inferior outcomes [52]. Likewise, in a recent BRPC cohort (*n* = 161), elevated pre- or peri-NAT CEA predicted failure to reach surgery under contemporary conversion protocols [53]. Overall, their use to assess treatment response after NAT remains exploratory and requires validation in larger, standardized cohorts.

***Practical takeaway***. After NAT, the decision to proceed to exploration should be made in sequence rather than on the basis of any single test. The first gate is conditional recovery: Patients should have adequate performance status, tolerate prior treatment, and retain sufficient nutritional reserve. The second gate is integrated oncologic reassessment. Contrast-enhanced CT and CA19-9 kinetics remain the pragmatic backbone of this evaluation. Although no universal CA19-9 threshold can be applied across all regimens and clinical contexts, normalization or a clear decline supports a favorable trajectory, whereas persistently rising or strikingly elevated post-NAT values should raise concern for adverse biology. Importantly, persistent vessel contact, stable radiographic appearance, or limited morphologic regression alone should not preclude exploration if metastatic progression is absent and the overall clinical–biologic profile is favorable.

## 6. When Is Staging Laparoscopy Still Useful After NAT?

After NAT, conversion to resection is consistently associated with longer survival in selected responders, provided that peritoneal, hepatic, and extra-regional nodal metastases have been excluded, a requirement that the NCCN recommends assessing by staging laparoscopy [8].

In the largest modern cohort, 1004 consecutive patients with radiographically localized PDAC underwent staging laparoscopy at Mayo Clinic (2017–2021): 18% had positive findings (gross metastases and/or positive cytology), but the rate was lower after NAT (14% vs. 22%). Importantly, a non-trivial yield persisted even after NAT, supporting routine consideration before laparotomy [54]. Complementing this, a multicenter cohort limited to resectable/BR PDAC disease found that staging laparoscopy still detected 10% occult metastases and more often redirected patients to systemic therapy rather than non-therapeutic laparotomy, supporting routine consideration [55]. In the post-NAT setting, even when the absolute yield is not necessarily higher than in treatment-naïve cohorts, staging laparoscopy may be particularly valuable for detecting interval metastatic progression before non-therapeutic laparotomy.

Beyond standard white-light staging laparoscopy, additional methods can support intraoperative staging in PDAC. Molecular peritoneal staging that detects mutant KRAS in peritoneal lavage during exploration using droplet digital PCR has shown feasibility and identifies biologically advanced disease even when conventional cytology is negative [56]. A larger prospective cohort measuring mutant KRAS ctDNA in both plasma and peritoneal fluid in localized PDAC stratified the risk of occult metastasis and poorer survival and was suggested to refine risk stratification when aggressive surgery is contemplated [57]. In parallel, indocyanine green (ICG) fluorescence imaging, an intraoperative adjunct that can enhance the visualization of target anatomy and vascular structures, has also been used to detect subcentimeter and subcapsular liver metastases from pancreatic cancer and can avert non-therapeutic laparotomy [58,59,60]. When combined with laparoscopic ultrasound, it shows additional potential to reveal otherwise occult hepatic deposits and to guide intraoperative decisions [61]. Contemporary expert consensus indicates that, although these techniques are promising for decision-changing detection after neoadjuvant therapy, standardization and PDAC-specific prospective validation are still needed before routine adoption [62].

***Practical takeaway***. After NAT, staging laparoscopy retains practical value because occult metastatic disease remains detectable despite apparently localized imaging. In this setting, its main role is to prevent non-therapeutic laparotomy and improve biologic selection before major resection. Molecular lavage assays, ICG imaging, and laparoscopic ultrasound are promising adjuncts, but they have not yet reached routine standard use.

## 7. How Should Vascular Involvement Be Managed at Exploration After NAT?

**Figure 1:**
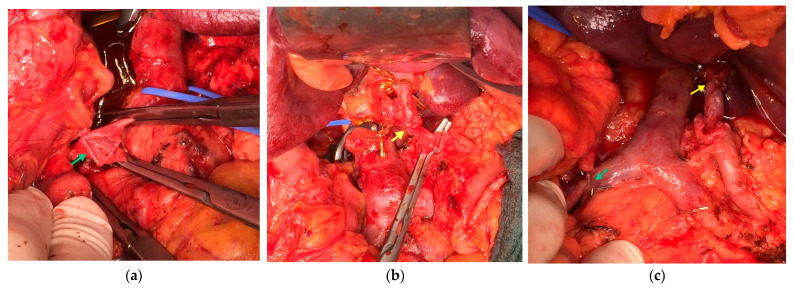
Intraoperative views of pancreatoduodenectomy (PD) with combined reconstruction of the superior mesenteric vein (SMV) and common hepatic artery (CHA). (**a**) SMV end-to-end anastomosis after segmental resection (green arrow). (**b**) CHA end-to-end anastomosis (yellow arrow). (**c**) Completion view showing both reconstructions in situ.

For patients proceeding to surgery following NAT, R0 resection (tumor-free margin > 1 mm) is recognized as a major determinant of outcome, and in BRPC/LAPC, this frequently entails the removal of intimately involved vascular structures [63]. Representative images from our institution illustrate combined venous (SMV) and arterial (CHA) resections with end-to-end reconstruction (Figure 1).

### 7.1. SMV/PV Resection (VR) and Reconstruction

In a 2011–2020 single-institution experience, the rate of VR among patients proceeding to resection was 28.6% for BRPC and 49.0% for LAPC (vs. 4.0% in anatomically resectable disease undergoing upfront surgery) [64]. VR is now an established adjunct to oncologic pancreatectomy, guided by the ISGPS position statement that standardizes indications and a practical four-type schema: primary repair/venorrhaphy, patch venoplasty, segmental resection with end-to-end anastomosis, and interposition grafting [65]. From a technical standpoint, the reconstruction strategy is primarily determined by the extent and geometry of venous involvement. Limited lateral wall involvement is generally amenable to tangential repair or patch venoplasty, whereas segmental resection is best followed by end-to-end anastomosis only when a tension-free approximation can be achieved; otherwise, interposition grafting becomes necessary. In practice, conduit choice should be individualized according to defect length, local contamination risk, venous anatomy, and center-specific experience [65,66].

Consistent with contemporary “benchmarking” across high-volume centers, pancreatoduodenectomy with VR can be performed with low in-hospital mortality (≤4%) and acceptable major morbidity (≤36%), while maintaining oncologic quality metrics—reinforcing the safety and feasibility of VR in expert settings. Moreover, outcomes vary with the specific resection and reconstruction performed. In the largest single-center European cohort to date (*n* = 2265 PDAC resections; 694 with VR), perioperative morbidity and portal vein thrombosis were highest after interposition grafting, whereas venorrhaphy/patch or end-to-end anastomosis had lower event rates; critically, when radical resection was achieved, median survival approached 23 months, underscoring that both thrombosis risk and surgical radicality shape prognosis [66]. Complementing these observations, propensity-matched and multivariable analyses indicate that the performance of VR itself—including the choice between tangential and segmental resection and the reconstruction method—is not independently associated with major morbidity, overall survival, recurrence-free survival, or locoregional recurrence; rather, outcomes are driven by tumor biology, patient factors, and the achievement of a margin-negative resection [64]. In the specific context of NAT, cohorts of surgical responders report similar survival with venous preservation compared with venous resection together with superior postoperative PV/SMV patency after preservation, which argues against routine VR when margin-negative clearance appears achievable without it [67]. Taken together, venous resection is a mature and standardized adjunct that is often essential to enable a true R0 resection after NAT in BRPC/LAPC, but its application should remain selective rather than routine.

### 7.2. Shunting and Bypass Strategies

**Figure 2:**
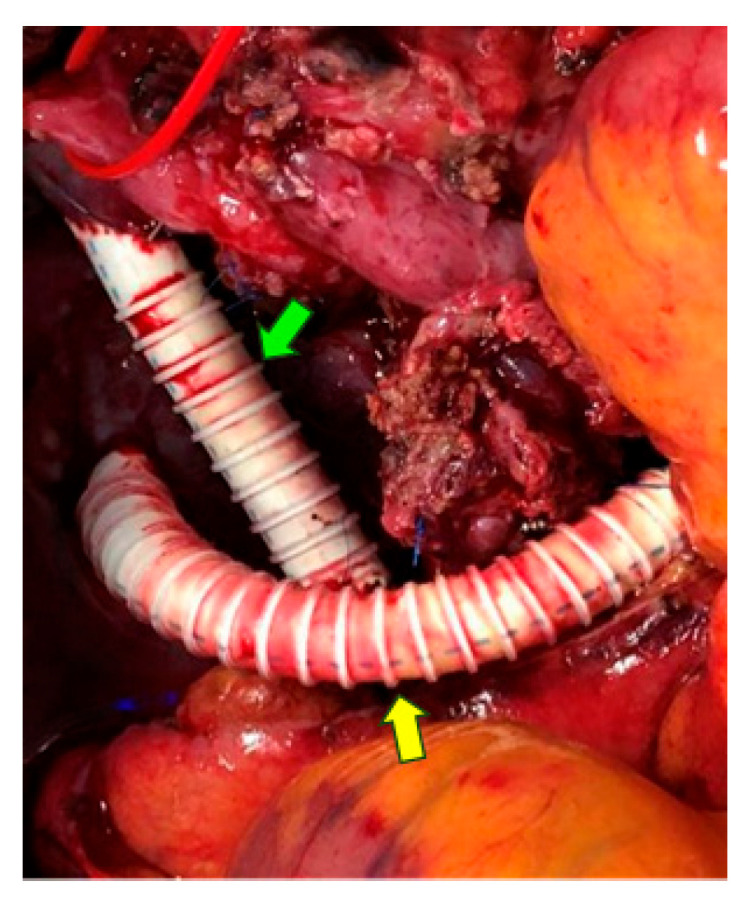
Complex venous reconstruction during pancreatectomy. Intraoperative view showing a dual-bypass approach to manage venous congestion. (1) Bypass 1 (green arrow): interposition graft between the portal vein (PV) and the superior mesenteric vein (SMV). (2) Bypass 2 (yellow arrow): systemic portal shunt connecting the SMV to the inferior vena cava (IVC) to alleviate mesenteric hypertension.

Technically, when a tumor extends over long segments of the portal–mesenteric venous axis and even involves jejunal or ileal branches, sometimes with cavernous transformation that disrupts mesenteric outflow, exposure, hemostasis, and reconstruction become particularly challenging. Current technique articles recommend organizing management around three complementary options: temporary intraoperative venous shunts or bypasses, definitive interposition grafting, and selective definitive portosystemic or splenorenal diversion in carefully chosen cases [68,69,70]. An intraoperative example of congestion-mitigation bypasses is shown in Figure 2, illustrating (1) a temporary bypass between the PV and SMV and (2) an alternative SMV-inferior vena cava bypass, used to maintain venous drainage during prolonged clamping and reconstruction.

The rationale for intraoperative shunting is to maintain hepatopetal inflow and prevent bowel edema, venous hypertension, and ischemia during periods of prolonged clamping or while grafts are harvested or thawed, and to stabilize venous return when chronic occlusion or cavernous collateralization precludes direct control of the portal vein [71,72]. A 2024 systematic review underscored that published experience remains limited, identifying only five studies with 145 patients in whom a shunt was created at pancreatectomy, most often during PD; within these data, the distal splenorenal shunt (DSRS) was the most frequently used shunt (about 76%), with mesoportal bypass (MPB) in roughly 10%, mesocaval shunt (MCS) in 11%, and combined DSRS plus MCS in 2%. Postoperative complications occurred in 44 of 145 patients (30%), and long-term patency of definitive shunts was 83% (110/120) [73]. More recently, a high-volume center reported 63 patients managed with temporary alloplastic venous bypass, including 34 MPB and 29 MCS; early outcomes were comparable between routes, and the choice between MPB and MCS was individualized to anatomy and anticipated arterial work [74]. Permanent MCS has also been described as an emergency or planned salvage option when graft-based reconstruction is not feasible in PD, again with acceptable early safety in selected cases [75,76]. At the other end of the spectrum, selected reports describe pancreatectomy with VR without reconstruction when robust collateral pathways can be preserved, underscoring that bypass or shunting should be individualized to venous anatomy and collateralization [77]. Accordingly, temporary MPB or MCS is most useful when prolonged clamping, chronic occlusion, or complex branch involvement threatens mesenteric drainage, whereas permanent shunting or splenorenal diversion should be reserved for selected salvage situations in which standard reconstruction is not feasible or when venous decompression is required.

### 7.3. Arterial Resection

Unlike the increasingly standardized approach to VR, major guidelines are cautious about AR. ESMO (2023) [11] states that arterial resection after induction therapy is generally not recommended, but may be considered case by case in experienced centers, whereas NCCN (v2.2025) [8] considers AR reasonable only in very select patients. The REDISCOVER International Consensus (2024) [78] is more specific, supporting consideration primarily for celiac axis and/or hepatic artery involvement, while not recommending routine SMA resection/reconstruction in the absence of consensus. Accordingly, AR should be pursued only when a clear oncologic benefit is anticipated, after MDT review, and by surgeons with dedicated vascular reconstruction expertise in high-volume centers [8,11,78]. In real-world practice, AR remains rare. In a nationwide Dutch cohort (2013–2019), 54 of 3868 pancreatectomies (1.4%) included AR, concentrated in a minority of centers and mostly planned procedures [79]; at a major European high-volume center, AR accounted for 39 of 3953 pancreatoduodenectomies (1.0%) over 2001–2019 [80].

AR during pancreatectomy carries a distinct risk profile. Early series reported substantial 90-day major morbidity and mortality, with deaths and severe complications predominantly from PPH, POPF, and ischemia [79,81]. An updated systematic review and meta-analysis (2018–2024) showed higher perioperative mortality with AR compared with non-arterial resections (≈three-fold), without a consistent increase in overall or major morbidity, and noted improving outcomes over time with centralization and experience [82]. With increasing centralization and accumulated technical experience, high-volume programs have reported improved perioperative outcomes, higher R0 resection rates, and better long-term results. In Heidelberg’s 385-case program, in-hospital mortality fell from 8.8% overall to 4.8% after 2013, with a learning curve of 15 ARs per surgeon identified on CUSUM analysis [83]. In a 236-procedure series, 30-day and 90-day mortality were 7.2% and 9.7%, respectively, with a program level inflection after 106 ARs and improved long-term outcomes in the most recent decade alongside broader NAT use [84]. Clearly, in the multimodal era of NAT plus surgery, surgeons should be prepared to meet the technical challenges of AR while preserving strict selection and centralization.

### 7.4. Artery Divestment Strategies

In parallel, artery-sparing strategies, including periarterial divestment (PAD), periadventitial dissection (often used synonymously with PAD and emphasizing dissection outside the arterial adventitia), and sub-adventitial divestment (within the plane between the adventitia and the external elastic lamina, EEL), have advanced as alternatives in the setting of post-NAT fibrosis; these planes and the EEL threshold are detailed in contemporary technique papers and reviews [85,86]. Early experience indicates that PAD can avoid AR with lower rates of POPF, PPH, ischemic complications, and relaparotomy, and in some cohorts a higher R0 resection rate, although the latter likely reflects earlier tumor stage and case selection rather than a causal effect of divestment [83,87]. In a recent head-to-head comparison for celiac axis involvement, DP with celiac axis divestment showed lower rates of POPF, intra-abdominal infection, and hepatic ischemia than en bloc AR with no significant differences in R0 rate, postoperative recurrence, or survival [88]. In a series of 125 BRPC and LAPC cases undergoing PAD, the 90-day mortality was 3.2%, the median overall survival was 20.6 months, and the one- and three-year survival rates were 73.2% and 22.9% respectively; the use of an arterial reinforcement patch (Neuro-Patch) on divested segments was independently associated with a lower rate of PPH (2% vs. 13.5%, *p* = 0.027) and may reduce pseudoaneurysm formation [89]. Indeed, larger and prospective datasets are needed to benchmark PAD against AR. For now, artery divestment should be viewed as a promising artery-sparing option in selected patients, particularly when a preserved periarterial or sub-adventitial plane suggests technical divestability without obvious full-thickness arterial invasion. If true arterial invasion is encountered intraoperatively, divestment should be abandoned, and the appropriateness of further radical resection must be reassessed according to technical feasibility and oncologic safety.

***Practical takeaway***. Vascular involvement at exploration after NAT should be managed selectively and in a margin-driven fashion rather than by routine en bloc resection. Portomesenteric venous resection is now an established adjunct when reconstruction is feasible, and in more complex long-segment or flow-compromised venous involvement, temporary bypasses or selective shunting strategies may be required to preserve mesenteric drainage and enable safe reconstruction. By contrast, arterial resection should remain highly restricted to carefully selected patients in expert centers. When the arterial plane is preserved, periarterial divestment may be considered; when true arterial invasion precludes safe divestment, surgeons must either proceed to formal arterial resection in an appropriate setting or abandon radical resection.

## 8. Frozen Section—When It Changes Management

Intraoperative FS is commonly used during pancreatectomy to confirm diagnosis and to guide management of modifiable transection margins, particularly at the pancreatic neck and bile duct, where further resection may be undertaken in an attempt to achieve an R0 resection [8]. However, evidence for oncologic benefit from FS-guided additional resection remains inconsistent [90]. A 2014 multicenter study including 1399 patients reported that converting a positive pancreatic neck FS to a negative margin by further resection was not associated with improved overall survival [91]. In contrast, a subsequent study found that complete tumor extirpation guided by FS, achieved either as en bloc resection or by revision resection, correlated with longer survival without an increase in perioperative morbidity or mortality [92]. Although NAT is often presumed to complicate margin assessment because fibrosis and treatment effect can challenge sampling and interpretation [93,94], recent data indicate that NAT does not reduce the diagnostic performance of FS for margin assessment [95]. In a multicenter cohort treated with NAT (*n* = 272), pursuing a negative neck margin after an initial positive FS has not conferred a survival advantage, which suggests that a positive FS may reflect adverse tumor biology rather than a remediable technical issue [96].

Evidence on the use of FS for intraoperative assessment of peri-vascular involvement is limited and shows modest diagnostic yield. In a single-center retrospective series of 85 pancreatectomies, targeted intraoperative FS from tissue abutting the PV/SMV, SMA, and CHA were benchmarked against the final medial surface of pancreas to adjudicate resectability. FS showed 38% sensitivity, 100% specificity, 100% positive predictive value, 58% negative predictive value, and 66% overall accuracy, with no false positives but a substantial false-negative rate, indicating that a negative peri-vascular FS cannot reliably exclude vascular encasement or invasion [97]. Technical descriptions of FS at the medial surface outline orientation and sampling strategies and also emphasize the difficulty of obtaining representative sections from fatty retroperitoneal tissue, underscoring the need for standardized handling [93,98]. In contemporary practice, consistent with the REDISCOVER recommendations and recent pathology reviews, a positive peri-vascular FS is treated as a decisive finding that should trigger vascular resection with reconstruction or, alternatively, the abandonment of radical surgery [78,99].

***Practical takeaway***. Frozen section after NAT should be used to guide targeted intraoperative decisions, not to justify unlimited revision resection. A positive peri-vascular frozen section is clinically meaningful and may support vascular resection with reconstruction or abandonment of radical surgery, whereas a negative result does not reliably exclude residual vascular involvement. In the NAT setting, margin positivity may reflect adverse biology as much as remediable technique.

## 9. Does NAT Change Postoperative Risk After Pancreatectomy?

Contemporary syntheses and large datasets consistently show that NAT does not worsen short-term surgical safety after pancreatectomy and, in selected settings, especially PD, is associated with fewer complications such as CR-POPF [100,101]. In an international analysis pooling 11,402 PDs for PDAC from North America and Europe, selection for NAT correlated with fewer CR-POPFs, fewer postoperative procedural interventions, and fewer infectious complications, with benefits persisting in sensitivity analyses of vascular resection cases [102]. Furthermore, recent studies suggest that the protective signal for POPF is more pronounced with chemoradiotherapy than with chemotherapy alone [103,104]. By contrast, for DP, a POPF-focused meta-analysis and a 2010–2023 propensity-matched international multicenter study both found no reduction in POPF with NAT and no differences in severe complications, reoperation, or 90-day mortality versus upfront surgery [105,106]. Notably, one large database analysis reported higher postoperative deep-vein thrombosis (DVT) after DP in patients receiving NAT, underscoring the need for vigilant thromboprophylaxis in this subgroup [107]. These findings indicate that NAT is perioperatively safe, confers complication reductions primarily in PD when chemoradiotherapy is used, leaves DP risk largely unchanged, and warrants regimen-specific selection with vigilant thromboprophylaxis for DP patients receiving NAT.

***Practical takeaway***. NAT does not appear to increase short-term surgical risk after pancreatectomy and may reduce selected complications, particularly CR-POPF after pancreatoduodenectomy. This benefit is less consistent for distal pancreatectomy, so postoperative risk assessment should remain procedure-specific.

## 10. How Should Anticoagulation Be Approached After Venous Reconstruction?

Antithrombotic management should be conceptualized in two layers: (i) routine postoperative VTE prophylaxis after major abdominal cancer surgery, and (ii) procedure- and reconstruction-specific strategies after PV/SMV resection/reconstruction, where both bleeding and thrombosis hazards may be amplified in the neoadjuvant era. There is no unified recommendation for routine anticoagulation after pancreatectomy, and most regimens are extrapolated from general cancer-associated thrombosis guidance rather than pancreas-specific trials [78,108]. International guidelines from ITAC 2022 [109], ESMO 2023 [110], and the ASCO update [111] advocate pharmacologic venous thromboembolism prophylaxis for hospitalized cancer patients and extended prophylaxis (up to 4 weeks) after major abdominopelvic surgery when bleeding risk is acceptable, but none provides pancreas- or reconstruction-tailored algorithms. Aligning with these oncology guidelines, the ERAS Society guideline for PD operation explicitly advises postoperative pharmacologic thromboprophylaxis and notes that, for high-risk oncologic patients, prophylaxis may be extended to four weeks [112]. The 2022 AHPBA document remains cautious, underscoring the lack of high-quality, pancreas-specific evidence and pointing out that several studies suggest early postoperative bleeding can outweigh thrombotic events, particularly in the immediate postoperative period [108]. Accordingly, individualized bleeding risk appraisal is central—especially when vascular resections/reconstructions are performed.

After venous resection, contemporary practice remains highly heterogeneous, with no consensus on whether to anticoagulate, when to start, or at what intensity, as demonstrated by an international survey spanning 65 centers across 17 countries [113]. A pancreas-specific systematic review of PV/SMV reconstruction that pooled 23 studies and 2751 patients demonstrated that thrombosis risk differs substantially by reconstruction method, being lowest after tangential repair or end-to-end anastomosis and highest with allogeneic or synthetic interposition grafts, while the marked heterogeneity of anticoagulation regimens precluded robust pooled estimates of any treatment effect [114]. For venous stenting after pancreatic surgery, a meta-analysis of 30 studies reported no improvement in patency with antithrombotic therapy compared with no antithrombotic therapy (antithrombotic group 22 studies, *n* = 207; no-antithrombotic group 8 studies, *n* = 61) and documented bleeding exclusively among anticoagulated patients, supporting selective rather than routine use [115]. In contrast, an international multicenter cohort including 972 patients who underwent venous resection observed a reduction in thirty-day PV thrombosis with early postoperative therapeutic anticoagulation, without a sustained benefit beyond ninety days; later thrombosis correlated most strongly with cancer recurrence [116]. Evidence for arterial resection is even more limited. In a single-center series of hepatic arterial resection and revascularization (*n* = 108), complete arterial occlusion occurred in 18% and outcomes did not differ by in-hospital anticoagulant choice or intensity, underscoring that pancreas-tailored anticoagulation algorithms remain unproven and should be individualized [117]. Procedure-stratified prospective studies are needed to determine the indications, timing, and intensity of antithrombotic therapy across defined settings, including benign versus malignant disease, the type of vascular resection, and the reconstruction technique.

***Practical takeaway***. Antithrombotic management after venous reconstruction should combine standard postoperative VTE prophylaxis with reconstruction-aware escalation when indicated. Because thrombosis risk varies substantially by reconstruction type, routine full anticoagulation for all patients is not supported by current evidence. Until pancreas-specific prospective data are available, therapy should be individualized according to reconstruction complexity, bleeding risk, and postoperative hemostatic stability.

## 11. Conclusions

NAT has reframed BR/LA PDAC as a biology-gated, time-sequenced surgical pathway. In BRPC, randomized evidence and guideline convergence support NAT as the default entry strategy, improving intention-to-treat oncologic endpoints and selecting against early systemic progressors. In LAPC, NAT enables conversion in a selected subset, but the apparent survival advantage of resection remains difficult to quantify because surgical cohorts are inherently selection-enriched—highlighting the need for randomized confirmation.

For surgeons, the priority is standardized decision-making. Operability after NAT should be gated first by physiologic recovery (performance status, toxicity resolution, nutrition/sarcopenia), then by integrated oncologic reassessment with contrast-enhanced CT and CA19-9 kinetics as the pragmatic backbone. Staging laparoscopy remains a practical safeguard against occult metastasis before high-stakes exploration. When exploration proceeds, vascular strategy should be margin-driven and selective: Portomesenteric venous resection is mature and reconstruction-dependent, whereas arterial resection should remain highly restricted to experienced centers, with divestment serving as an artery-sparing alternative under strict stop rules.

Future progress will depend on procedure-stratified prospective studies that harmonize definitions, prespecify restaging thresholds, and test reconstruction-specific antithrombotic strategies.

## Figures and Tables

**Table 1:** Guideline recommendations for neoadjuvant therapy in BRPC and LAPC.

	BRPC	LAPC
NCCN v2.2025 [8]	Recommended: (m)FOLFIRINOX/GnP ± subsequent CRT are preferred ^1^.	Clinical trial preferred. NAT can be selected: (m)FOLFIRINOX/GnP/NALIRIFOX + CRT/SBRT preferred ^1^.
ESMO 2023/2024 [11]	Recommended when trial enrollment is not feasible: FOLFIRINOX/GnP ± subsequent CRT suggested.	Recommended when trial enrollment is not feasible. FOLFIRINOX/GnP can be chosen.
JPS 2023 [14]	Recommended: no regimen suggestion	Initial systemic therapy is recommended. FOLFIRINOX/GnP/Gem/S-1 recommended; following resection is one of the treatment options.
CSCO 2022 [15]	Clinical trials are preferred. NAT is recommended.	Clinical trials are recommended; NAT can be chosen.
ASCO 2016/2019 [12,13]	Recommended: no regimen suggestion	Initial systemic therapy is recommended; curative resection is rare.

NAT, neoadjuvant therapy; BRPC, borderline resectable pancreatic cancer; LAPC, locally advanced pancreatic cancer; NCCN, National Comprehensive Cancer Network; ESMO, European Society for Medical Oncology; JPS, Japanese Pancreas Society; CSCO, Chinese Society of Clinical Oncology; ASCO, American Society of Clinical Oncology; CRT, chemoradiotherapy; SBRT, stereotactic body radiotherapy; FOLFIRINOX, 5-fluorouracil (5-FU) + leucovorin + irinotecan + oxaliplatin; mFOLFIRINOX = modified-dose FOLFIRINOX; GnP, gemcitabine + nab-paclitaxel; NALIRIFOX, liposomal irinotecan + 5-FU/leucovorin + oxaliplatin; S-1, oral fluoropyrimidine (tegafur/gimeracil/oteracil). ^1^ FOLFIRINOX/mFOLFIRINOX/GemCis ± subsequent CRT are preferred for known BRCA1/2 or PALB2 mutations.

## Data Availability

No new data were created or analyzed in this study.
